# Intrarenal epidermoid cyst mimicking a cystic renal cell carcinoma: A case report and review of literature

**DOI:** 10.1002/ccr3.9086

**Published:** 2024-06-11

**Authors:** Mohammadhossein Khorraminejad‐Shirazi, Seyed Ali Nabavizadeh, Babak Shirazi Yeganeh, Maryam Ahmadifar, Mehdi Salehipour, Seyed Hamed Jafari, Navid Omidifar

**Affiliations:** ^1^ Department of Pathology, School of Medicine Shiraz University of Medical Sciences Shiraz Iran; ^2^ Student research committee Shiraz University of Medical Sciences Shiraz Iran; ^3^ Department of Pathology, School of Medicine Jahrom University of Medical Sciences Jahrom Iran; ^4^ Department of Otolaryngology, Otolaryngology Research Center Shiraz University of Medical Sciences Shiraz Iran; ^5^ Department of Urology, School of Medicine Shiraz University of Medical Sciences Shiraz Iran; ^6^ Medical Imaging Research Center Shiraz University of Medical Sciences Shiraz Iran

**Keywords:** cystic renal mass, epidermoid cyst, pathology, radical nephrectomy

## Abstract

**Key Clinical Message:**

This case highlights the diagnostic pitfalls that can occur when evaluating complex cystic renal masses. Distinguishing epidermoid cysts from renal cell carcinoma is difficult but imperative to guide conservative management when appropriate, avoiding unnecessary nephrectomy.

**Abstract:**

Renal epidermoid cysts are extremely rare, with only 12 cases reported in the literature. Their radiographic features often resemble cystic renal cell carcinoma, frequently prompting unnecessary nephrectomy. A 64‐year‐old man with a history of nephrolithiasis presented with left flank pain and hematuria. Imaging revealed a complex cystic renal mass suspicious for renal cell carcinoma. Following left radical nephrectomy, histopathology examination revealed a benign epidermoid cyst. Renal presentation of epidermoid cyst poses unique diagnostic and therapeutic challenges. Possible pathogenesis includes ectopic epidermal implantation during embryogenesis or squamous metaplasia following chronic irritation or deficiency. Radiographic distinction from concerning entities like renal cell carcinoma is difficult but imperative to avoid extensive surgery. This case highlights the diagnostic pitfalls and management considerations for renal epidermoid cysts. Additional study of clinical and imaging factors that distinguish epidermoid cysts from renal cell carcinoma can guide conservative management when appropriate, avoiding unnecessary nephrectomy for benign disease.

## 1 INTRODUCTION

Epidermoid cysts are common cutaneous lesions but remarkably rare within the kidney. Their renal presentation poses unique diagnostic and therapeutic challenges.^1^, ^2^ Radiographically, they often resemble more neoplastic processes like cystic renal cell carcinoma, frequently prompting unnecessary nephrectomy.^2^ Despite concerning imaging features, epidermoid cysts remain benign.^3^

Epidermoid cysts and dermoid cysts represent distinct pathological entities, despite sharing some similarities. Epidermoid cysts are benign cystic lesions lined by stratified squamous epithelium and containing purely desquamated keratin material. In contrast, dermoid cyst contains pilosebaceous appendages like hair follicles, sweat glands, and sebaceous glands in addition to the keratinizing squamous coating.^2^ Epidermoid and dermoid cysts can infrequently develop in atypical sites like the round ligament, sublingual region, bladder, and even within organs like the kidney.^4^, ^5^, ^6^, ^7^, ^8^

Their rarity, with only 12 documented in the literature, renders their precise pathogenesis and origin in the kidney unclear. Accurate diagnosis is imperative to avoid extensive surgery and preserve renal function. Here, we present a case of a renal epidermoid cyst, accompanied by a comprehensive literature review to further characterize these uncommon intrarenal cysts.

## 2 CASE HISTORY

A 64‐year‐old man with a past medical history of hypertension, horseshoe kidney, and two prior open nephrolithotomies presented with left flank pain radiating to the left lower quadrant of abdomen and microscopic hematuria.

**FIGURE 1:**
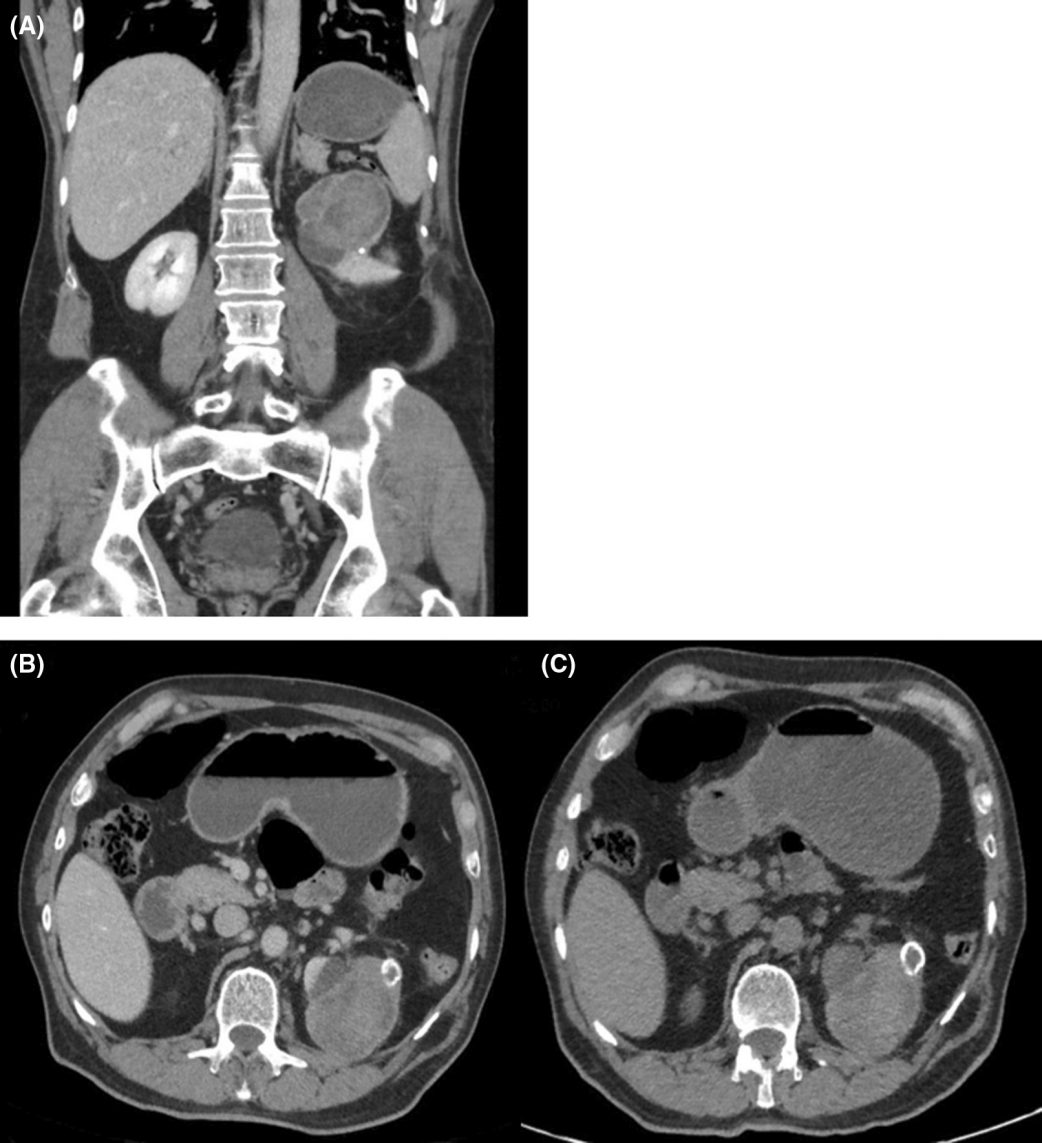
(A) Coronal contrast‐enhanced CT image demonstrates a large, lobulated cystic mass in the upper pole of the left kidney. (B, C) Axial contrast‐enhanced CT (B) and non‐contrast CT (C) image shows a well‐circumscribed cystic lesion in the upper pole of the left kidney.

The physical examination was unremarkable. Computed tomography (CT) imaging demonstrated a 60 × 60 × 55mm lobulated cystic lesion in the upper pole of the left kidney, with foci of calcification and heterogeneous density. No measurable enhancement was seen, although mild thickening and enhancement of the cyst wall were noted (Figure 1).

Given radiographic suspicion for a cystic renal cell carcinoma, the patient underwent a left radical nephrectomy.

## 3 METHODS

**FIGURE 2:**
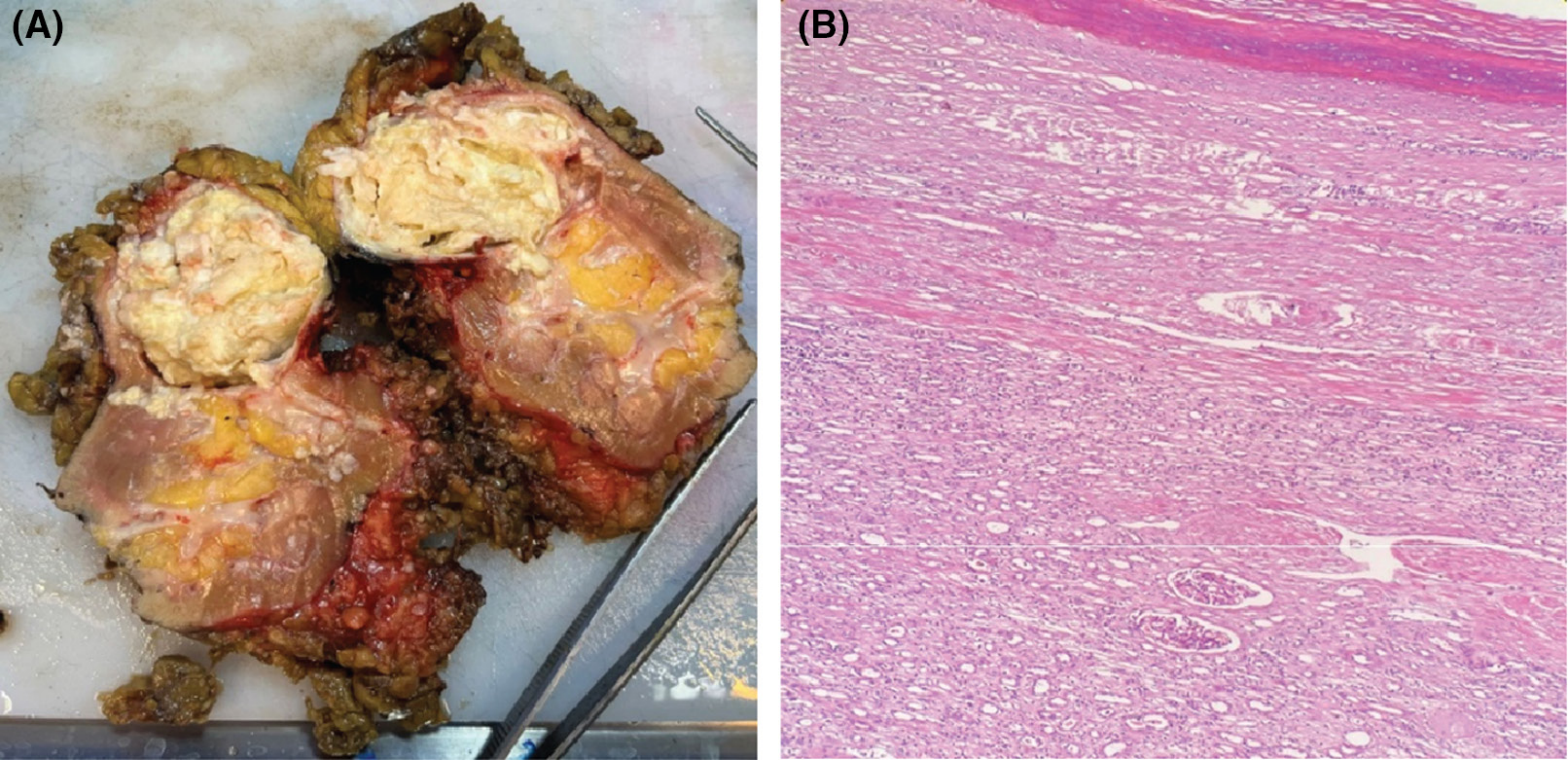
(A) Gross specimen shows cystic lesion arising from the upper pole of the kidney. The mass has a smooth, glistening outer surface and measures approximately 6 cm in greatest dimension. The cyst was filled with soft and waxy yellowish material. (B) Microscopic image, H&E stain, 40× magnification. The cyst is lined by stratified squamous epithelium with a granular cell layer, filling the cystic space. Underneath the squamous epithelium lining, glomeruli and tubules can be noticed.

Gross pathological examination of the nephrectomy specimen revealed a well‐circumscribed cystic structure protruding from the kidney parenchyma. Sectioning displayed a unilocular cyst filled with soft and waxy yellowish material. Microscopic analysis demonstrated a cyst wall lined by stratified squamous epithelium with a granular layer, with no atypia, and filled with laminated keratin debris (Figure 2). To differentiate between an epidermoid cyst and a dermoid cyst, the entire cyst wall was embedded and examined microscopically. It did not show any skin appendages, consistent with the diagnosis of an epidermoid cyst.

## 4 CONCLUSION AND RESULTS

The patient had an uncomplicated postoperative course.

## 5 DISCUSSION

In this article, we report a case of a 64‐year‐old man with a history of prior nephrolithotomies who presented with left flank pain and microscopic hematuria. The imaging characteristics and clinical presentation strongly suggested a malignant cystic neoplasm that was followed by radical nephrectomy. As illustrated in Table 1, the main symptom in these documented cases is flank pain, which is not very distinctive, making the condition difficult to diagnose.^2^, ^9^, ^10^, ^11^, ^12^

**TABLE 1:** Reported cases of renal epidermoid cyst.

Author/Year	Age/Sex	Tumor location	Sign and symptoms	Kind of surgery	Outcome	Intrarenal OR renal pelvis
Present case	64/M	Upper pole of left kidney	Left flank pain, radiating pain to left lower quadrant, microscopic hematuria	Left radical nephrectomy	Uneventful recovery	Intrarenal
Ademayali Ido et al. 2023^2^	45/F	intra parenchymatous mass with irregular contours	Right flank pain with dysuria and macroscopic hematuria for 6 months	Right radical nephrectomy	Uneventful post‐surgical recovery after one year	Intrarenal
Barrios Barreto et al., 2021^1^	56/M	Upper right pole at the pyelocaliceal level	Lumbar pain, macroscopic hematuria, dysuria	Partial nephrectomy of the upper renal pole by laparoscopy	Uneventful recovery	Renal pelvis
Pradhan et al., 2017^18^	62/F	Lower pole of the left kidney	Bilateral renal cysts, recurrent UTIs, microhematuria, 6.5 cm mass in left kidney	Laparoscopic radical nephrectomy	Disease free for the last year, uneventful post‐surgical follow‐up	Intrarenal
Go et al., 2012^14^	73/F	Renal pelvis of left kidney	Incidentally detected renal pelvis mass; no episodes of renal colic or urolithiasis; no tenderness on the area of costovertebral angle	Total nephroureterectomy	Discharged without postoperative complications	Renal pelvis
Desai et al., 2011^13^	74/M	Upper pole of right kidney	Painless hematuria	Radical nephrectomy	Uneventful recovery	Intrarenal
Dadali et al., 2010^3^	50/F	Right kidney	Lumbar pain, hematuria, dysuria	Laparoscopic transperitoneal nephrectomy	Uneventful recovery	Intrarenal
Abdou and Asaad, 2010^9^	67/M	Left kidney	Left flank pain	Left nephrectomy	Uneventful recovery	Intrarenal
Lim and Kim, 2003^12^	51/M	Lower pole of left kidney	Left flank pain, gross hematuria	Left retroperitoneal laparoscopic simple nephrectomy	Uneventful recovery	Intrarenal
Gokce et al., 2003^11^	55/F	Middle and lower calyces, extending to renal pelvis	Right flank pain, gross hematuria	Right radical nephroureterectomy	Uneventful recovery	Renal pelvis
Emtage and Allen, 1994^10^	74/F	Middle third of right kidney	Right flank pain	Right radical nephrectomy	Uneventful recovery	Intrarenal
Duprat et al., 1986^17^	4/M	Upper pole of right kidney	Urinary frequency	Right radical nephrectomy	Uneventful post‐op recovery	Intrarenal
Krogdahl, 1979^19^	67/M	Upper pole of left kidney	Left renal colic, repeated attacks, dysuria, pollakiuria	Partial nephrectomy	Complete tumor removal, uneventful recovery	Intrarenal

The pathogenesis of epidermoid cysts of the kidney remains controversial, with several theories proposed to explain their histogenesis. One prevailing theory suggests that these cysts are formed due to atypical ectodermal placement during the embryonic stage, particularly from residual Wolffian duct tissues.^2^ According to this perspective, the misplaced ectodermal cells contribute to the presence of epidermal tissue fragments within the renal architecture, which subsequently evolve into epidermoid cysts.^3^, ^13^ Moreover, squamous metaplasia has been proposed as the etiology of renal epidermoid cysts. This pathological change can be of traumatic or deficiency origin.^1^ On one hand, the traumatic origin is associated with prolonged irritation induced by renal stones and by treatments like shock wave lithotripsy.^12^ On the other hand, the deficiency origin is linked to vitamin A deficiency, which is known to induce keratinizing squamous metaplasia in the urothelium.^1^, ^2^, ^14^ As mentioned in our case presentation, our patient did not have any history of trauma but did have a prior history of nephrolithotomy. Given this clinical context, the possible pathogenesis and etiology of the epidermoid cyst of our case could be attributed to chronic irritation and squamous metaplasia secondary to nephrolithiasis.

The radiographic evaluation of renal epidermoid cysts poses a diagnostic challenge due to their similar features with other renal conditions.^2^ Epidermoid cysts of the kidney typically appear on imaging as cystic lesions with irregular contours and possible stippled or coarse calcifications in the cyst wall or lumen, thus they can mimic more concerning renal masses like renal cell carcinoma.^1^ Radiographically, differentiation between epidermoid and dermoid cysts on MRI can be facilitated by observing signal intensities; typically, epidermoid cysts exhibit hyperintense signals on diffusion‐weighted imaging (DWI) and are hypointense on T1‐weighted images, whereas dermoid cysts generally display hyperintense signals on T2‐weighted images due to their higher fat content.^2^, ^15^

Other key entities in the differential diagnosis of a cystic renal mass with calcification include teratomas, tuberculomas, Wilms tumors, xanthogranulomatous pyelonephritis, and osteogenic sarcoma metastases.^3^, ^16^, ^17^ Correlation with clinical findings can help distinguish epidermoid cysts from more worrisome renal masses when the imaging appearance is equivocal.

In conclusion, we present a challenging case of an epidermoid cyst that was diagnosed as renal cell carcinoma based on concerning radiographic features. This case highlights the diagnostic pitfalls that can occur when evaluating complex cystic renal masses. Additional study is imperative to identify imaging and clinical factors that can help distinguish these entities, guiding appropriate management. This can facilitate conservative management when appropriate and avoid extensive surgery for benign disease.

## AUTHOR CONTRIBUTIONS

**Mohammadhossein Khorraminejad‐Shirazi:** Methodology; writing – review and editing. **Seyed Ali Nabavizadeh:** Methodology; writing – original draft; writing – review and editing. **Babak Shirazi Yeganeh:** Writing – original draft; writing – review and editing. **Maryam Ahmadifar:** Writing – review and editing. **Mehdi Salehipour:** Writing – review and editing. **Seyed Hamed Jafari:** Methodology; writing – review and editing. **Navid Omidifar:** Methodology; writing – review and editing.

## FUNDING INFORMATION

No source of funding.

## CONFLICT OF INTEREST STATEMENT

The authors declare that they have no competing interests.

## ETHICS STATEMENT

The present study was approved by the Medical Ethics Committee of Shiraz University of Medical Sciences (#29597). The purpose of this report was completely explained to the patient and written inform consent was obtained from the patient.

## CONSENT

Written informed consent was obtained from the patient to publish this report in accordance with the journal'spatient consent policy.

## Data Availability

Data of the patient can be requested from authors. Please write to the corresponding author if you are interested in such data.
